# Effects of juglone and lawsone on oxidative stress in maize coleoptile cells treated with IAA

**DOI:** 10.1093/aobpla/plw073

**Published:** 2016-11-17

**Authors:** Renata Kurtyka, Wojciech Pokora, Zbigniew Tukaj, Waldemar Karcz

**Affiliations:** 1 Department of Plant Physiology, Faculty of Biology and Environmental Protection, University of Silesia, Jagiellońska 28, PL-40 032 Katowice, Poland; 2 Department of Plant Physiology and Biotechnology, Faculty of Biology, University of Gdańsk, Wita Stwosza 59, PL-80 308 Gdańsk, Poland

**Keywords:** Coleoptile segments, juglone, lawsone, oxidative stress, *Zea mays*

## Abstract

Naphthoquinones are secondary metabolites widely distributed in nature and produced by bacteria, fungi and higher plants. Their biological activity may result from induction of oxidative stress, caused by redox cycling or direct interaction with cellular macromolecules, in which quinones act as electrophiles. The redox homeostasis is known as one of factors involved in auxin-mediated plant growth regulation. To date, however, little is known about the crosstalk between reactive oxygen species (ROS) produced by quinones and the plant growth hormone auxin (IAA). In this study, redox cycling properties of two naphthoquinones, juglone (5-hydroxy-1,4-naphthoquinone) and lawsone (2-hydroxy-1,4-naphthoquinone), were compared in experiments performed on maize coleoptile segments incubated with or without the addition of IAA. It was found that lawsone was much more effective than juglone in increasing both H_2_O_2_ production and the activity of antioxidative enzymes (SOD, POX and CAT) in coleoptile cells, regardless of the presence of IAA. An increase in the activity of Cu/Zn-SOD isoenzymes induced by both naphthoquinones suggests that juglone- and lawsone-generated H_2_O_2_ was primarily produced in the cytosolic and cell wall spaces. The cell potential to neutralize hydrogen peroxide, determined by POX and CAT activity, pointed to activity of catalase as the main enzymatic mechanism responsible for degradation of H_2_O_2_. Therefore, we assumed that generation of H_2_O_2_, induced more efficiently by LW than JG, was the major factor accounting for differences in the toxicity of naphthoquinones in maize coleoptiles. The role of auxin in the process appeared negligible. Moreover, the results suggested that oxidative stress imposed by JG and LW was one of mechanisms of allelopathic action of the studied quinones in plants.

## Abstract (precis)

Despite the large number of papers that have been published on juglone and lawsone cytotoxicity, little is known about their redox cycling properties in plants. Here, the effects of these two naturally occurring naphthoquinones on H2O2 production and the activity of ROS scavenging enzymes (SOD, POX, CAT) in maize coleoptile segments were measured. It was found that lawsone was a more effective oxidative stress inducer in maize coleoptile cells than juglone. The results suggested that oxidative stress imposed by juglone and lawsone was one of the mechanisms of allelopathic action of the studied quinones in plants.

## Introduction

Juglone (JG) (5-hydroxy-1,4-naphthoquinone) and lawsone (LW) (2-hydroxy-1,4-naphthoquinone) are naturally occurring naphthoquinones that are widespread in nature. They have been identified as secondary metabolites that can be isolated from the leaves, roots, husks and bark of plants of the *Juglandaceae* family, particularly black walnut (*Juglans nigra*) in case of JG, and from the leaves of the henna plant (*Lawsonia inermis*) and water hyacinth (*Eichhornia crassipes*) in case of LW (Solar *et al.* 2006; Babula *et al.* 2009; Ashnagar and Shiri 2011; Nour *et al.* 2013). Juglone has varying effects on plants, including an inhibition of seed germination and plant growth (Hejl and Koster 2004; Böhm *et al.* 2006; Sytykiewicz 2011; Babula *et al.* 2014; Rudnicka *et al.* 2014), a reduction in the chlorophyll content (Terzi *et al.* 2003), a disruption of the root plasma membrane and a decrease in water uptake (Hejl and Koster 2004), as well as inhibition of photosynthesis (Hejl *et al.* 1993; Jose and Gillespie 1998), respiration (Jose and Gillespie 1998; Hejl and Koster 2004; Babula *et al.* 2009), transpiration (Jose and Gillespie 1998) and stomatal conductance (Jose and Gillespie 1998). In experiments performed on tobacco BY-2 cells, Babula *et al.* (2009) showed the ability of juglone to generate reactive oxygen species (ROS) and suggested that these substances play an important role in processes of programmed cell death. Additionally, in studies carried out on lettuce seedling roots, juglone caused enhanced production of H_2_O_2_, followed by a significant increase in the amount of free intercellular calcium ions in both cortical and peripheral cells of the root cap (Babula *et al.* 2014). JG and LW display a related chemical structure (Kumagai *et al.* 2012), however, knowledge about the effects of LW in plants is limited and the main area of LW research has been its interaction with animal and human tissues (Öllinger and Brunmark 1991; Kumbhar *et al.* 1996; Dasgupta *et al.* 2003).

One of the most important mechanisms underlying the phytotoxic influence of JG and LW is associated with their strong redox activity, which is involved in the peroxidation action within the tissues of targeted plants (El Hadrami *et al.* 2005; Chobot and Hadacek 2009; Ashnagar and Shiri 2011; Hao *et al.* 2012). On the other hand, some studies have demonstrated the protective role of juglone and lawsone, which has been observed as an abatement of oxidative stress and an inhibition of macromolecular oxidation (Chobot and Hadacek 2009; Chi *et al.* 2011; Cheniany *et al.* 2013).

Oxidative stress refers to the uncontrolled production of reactive oxygen species (ROS) such as superoxide anion (O_2_^.−^), hydrogen peroxide (H_2_O_2_), hydroxyl radical (^.^OH) and singlet oxygen (_1_O^2^). Every cell has various mechanisms, both non-enzymatic and enzymatic, to regulate the ROS level such as superoxide dismutases (SODs), catalases (CATs) and peroxidases (POXs) (recently reviewed in Kärkönen and Kuchitsu 2015). In plant cells, superoxide dismutases act as the first line of defence against ROS. SODs are classified into three groups according to their metal cofactor: copper-zinc (Cu/Zn-SOD), manganese (Mn-SOD) and iron (Fe-SOD) (Alscher *et al.* 2002). Unlike other organisms, plants have multiple SOD forms, i.e. one type of SOD can be present in several isoforms that have the same catalytic specificity, but have different kinetic proprieties and different migration rates on a gel (Kephart 1990). SODs catalyse the reaction of a disproportionation of two molecules of a superoxide radical ion to an oxygen molecule and H_2_O_2_ molecule, which is then further scavenged by catalases and peroxidases. Since biological membranes are impermeable to most of the ROS, SOD isoforms that occur at sites where O_2_^.−^ is generated and play a role in the neutralisation of ROS in all cellular compartments, the cytosol and apoplastic spaces, mitochondria as well as in the water–water cycle and the ascorbate–glutathione cycle in chloroplasts (Asada 1999). Most of the H_2_O_2_ that is generated via SOD activity is then scavenged by peroxisomal and glyoxysomal catalases, while its molecules, which are unavailable for catalases, are degraded by peroxidases due to their high affinity to H_2_O_2_ and their presence in other cell compartments (Mittler 2002).

There is a close interdependence among the activity of SOD, CAT and POX because the product of the reaction that is catalysed by SOD becomes a substrate for POX and CAT. Moreover, the intermediates of the superoxide radical anion reduction to an oxygen molecule play a role in a number of regulatory and signalling processes. It is believed that H_2_O_2_ plays a key role in the regulation of the expression of antioxidative enzymes (SOD, POX, CAT) as well as other defence proteins such as pathogenesis-related proteins and heat shock proteins (HSPs) (Knight and Knight 2001).

The main aim of the present study was to compare the effects of two naturally occurring naphthoquinones, juglone (5-hydroxy-1,4-naphthoquinone) and lawsone (2-hydroxy-1,4-naphthoquinone), on H_2_O_2_ production and the activity of ROS scavenging enzymes (SOD, POX, CAT). In addition, the effect of auxin (IAA) on these processes was also studied. To the best of our knowledge, no research has been reported on the interdependence between oxidative stress induced by both naphthoquinones and the effects of plant growth hormone auxin (IAA). The experiments were performed with segments of etiolated maize coleoptiles, which are a classical model system for studies on the mechanism of auxin action on plant cell growth. In this system, the number of cells is constant and an organ grows only through elongation. Interestingly, most of the important evidence on the molecular mechanisms of auxin action was obtained in experiments performed with segments of maize coleoptiles (reviewed in Hager 2003). Despite the large number of papers that have been published on juglone and lawsone cytotoxicity, there is still a lack of a clear explanation of their redox cycling properties in plant cells. Moreover, our experiments may provide new data on the allelopathy of both naphthoquinones and the possible role of IAA in this process.

## Methods

### Plant material

Caryopses of maize (*Zea mays* cv. Cosmo) were soaked in tap water for 2 h, sown on wet wood wool in plastic boxes and were placed in a growth chamber for 4 days (Type MIR-553, Sanyo Electric Co., Japan) at 27 ± 1 °C. The experiments were performed with 10-mm-long coleoptile segments cut from etiolated seedlings. The coleoptile segments, with the first leaves removed, were excised 3 mm below the tip and incubated in a control medium of the following composition: 1 mM KCl, 0.1 mM NaCl, 0.1 mM CaCl_2_, initial pH 5.8–6.0.

Before chemical analysis, 20 intact coleoptile segments were placed in a measuring chamber and pre-incubated for 2 h in 6.0 cm^3^ (0.3 cm^3^ segment^−^^1^) of an intensively aerated control medium. After the pre- incubation of the coleoptile segments, juglone (5-hydroxy-1,4-naphthoquinone) (50 µM) or lawsone (2-hydroxy-1,4-naphthoquinone) (100 µM), with or without the addition of indole-3-acetic acid (IAA) (100 μM), were introduced into the incubation medium for 4 h. The concentrations of juglone and lawsone (50 and 100 µM, respectively) were selected in accordance with our previous studies, based on dose–response curves plotted for the effects of juglone and lawsone on endogenous and IAA-induced growth of maize coleoptile segments. In these experiments, the above-mentioned concentrations of juglone and lawsone inhibited both endogenous and IAA-induced growth of maize coleoptile segments by *ca.* 50 % (Rudnicka *et al.* 2014, 2015). Moreover, the experimental conditions such as number of coleoptiles per medium volume, composition of the incubation medium and initial pH of the medium were also adopted from our previous growth experiments (Karcz and Burdach 2002; Kurtyka *et al.* 2011, 2012; Burdach *et al.* 2014; Rudnicka *et al.* 2014). All experiments were performed in the time interval of 6 h, covering the time course of the model curve proposed by Becker and Hedrich (2002) for IAA-induced growth of maize coleoptile segments. On the other hand, our preliminary experiments also evidenced that the 6 h period is long enough to reveal differences between the action of both naphthoquinones. For the detection of H_2_O_2_ and the enzyme assay, samples were collected at 15, 60 and 120 min of the pre-incubation in the control medium and at 135, 180, 240, 300 and 360 min of the incubation in the mediums with IAA, JG and LW. All manipulations and experiments were conducted under a dim green light (0.04 W m^−2^), impinging omnilaterally on the coleoptiles.

### Chemicals

Juglone (5-hydroxy-1,4-naphthoquinone) and lawsone (2-hydroxy-1,4-naphthoquinone) were obtained from Sigma Aldrich Inc. (St. Louis, MO, USA). The naphthoquinones, juglone and lawsone were dissolved in deionised water and used at a final concentration of 50 and 100 µM, respectively.

An aqueous stock solution (1 mM) of indole-3-acetic acid (IAA) (Serva, Heidelberg, Germany) was prepared with the potassium salt of IAA. IAA was used at a final concentration of 100 µM.

### Hydrogen peroxide detection

The concentration of H_2_O_2_ was assayed using an Amplex^®^UltraRed fluorochrome (Molecular Probes). An aliquot of 100 µl of suspension that was withdrawn from the vessel in which the coleoptiles were incubated was immediately placed in a 96-well plate and 10 µl of a 100 µmol solution of Amplex^®^UltraRed in a HEPES buffer (pH 7.0, 0.05 M) was added. After incubating the plate for 20 min at 30 °C in darkness, the fluorescence was measured at Ex/Em wavelengths 490 nm/585 nm using a Varioskan^®^ Flash Spectral Scanning Multimode Microplate Reader spectrofluorometer (ThermoScientific) running Varioskan Flash SkanIt Software (2006). The ‘blind’ probes, which contained a 110 µl of 10 µmol solution of Amplex^®^UltraRed in a HEPES buffer (pH 7.0, 0.05 M) or 100 µl of the coleoptile incubation buffer with 10 µl of a HEPES buffer, were measured to subtract autofluorescence of the fluorochrome or coleoptile incubation buffer, respectively. The standard curve was prepared using the stabilised H_2_O_2_ solution (Sigma Aldrich, Germany), which was diluted with a HEPES buffer (pH 7.0, 0.05 M) to a concentration range of 0.1–100 µmol. The concentration of H_2_O_2_ is presented as nmol of H_2_O_2_ per mg of the fresh weight of a coleoptile.

### Enzyme isolation and assay

For protein isolation, maize coleoptiles (20 pcs.) were placed in 15 ml capacity test tubes with a portion of glass beads (∼0.5 g) and suspended in 0.5 ml of an extraction buffer. In order to isolate superoxide dismutase and catalase 0.05 M of a potassium phosphate buffer (pH 7.8) containing protease inhibitors (0.01 M EDTA, 0.01 M E-64 (*trans*epoxysuccinyl-l-leucylamido(4-guanidino)butane), 0.01 M DTT (DL-dithiothreitol) were used and for peroxidase: 50 mM of a phosphate buffer 7.6, 0.86 mM AsA (ascorbic acid), 2.4 mM EDTA (ethylenediaminetetraacetic acid), 20 % glycerol and 2 % PVP (polyvinylpyrrolidone) were used. The coleoptiles were homogenised with glass beads in an MP Bio homogeniser (45 s, 5000 rps). The homogenate was transferred into an Eppendorf tube and the glass beads were washed three times with 0.5 ml of the appropriate buffer to a final volume of 2 ml. Debris was removed by centrifugation (20 min, 20 000 *g*, 4 °C). The supernatant that was obtained was used for the enzyme assays. The protein content in the supernatant was evaluated according to the method of Bradford (1976).

Total SOD activity was assayed following Beauchamp and Fridovich (1971). The reaction mixture contained 2.4 × 10^−^^6^ M riboflavin, 0.01 M methionine and 1.67 × 10^−^^6^ M nitroblue tetrazolium (NBT) in a 0.05 M potassium phosphate buffer, pH 7.8. Afterwards, 2.5 ml of the reaction mixture and 0.5 ml of the crude extract was illuminated in glass tubes for 10 min with white fluorescent light at an intensity of 60 Wm^−^^2^. The absorbance was measured at 560 nm with an illuminated 2.5 ml of the reaction mixture and 0.5 ml of a potassium phosphate buffer as a control. An SOD activity unit was defined as the amount that caused a 50 % inhibition of NBT reduction. Results are given as units of SOD activity per mg of protein (U mg^−^^1^).

The activity of SOD isoforms was determined using an indirect method that involved the inhibition of NBT as described by Tukaj and Pokora (2006). Briefly, non-denaturating PAGE was performed according to Davies (1971) with the modification that both the stacking (4.5 %) and resolving (12.5 %) gels were polymerised with riboflavin (2.8 × 10^−^^5^ M). Each sample contained 50 µg of the protein. SODs were located on the gels according to Beauchamp and Fridovich (1971). The gels were soaked in 2.45 × 10^−^^3^ M NBT for 20 min, followed by immersion (15 min) in a solution containing 0.028 M TEMED and 2.8 × 10^−^^5^ M riboflavin in a 0.036 M potassium phosphate buffer, pH 7.8. The gels were illuminated until they were uniformly blue and the sites where SOD was present were achromatic. SOD isoforms were identified according to their different sensitivities to H_2_O_2_ (5 mM) and KCN (2 mM). Cu/Zn-SOD was inhibited by both CN^−^ and H_2_O_2_, Fe-SOD was resistant to CN^-^ and sensitive to H_2_O_2_, while Mn-SOD was resistant to both chemicals. The activity of a separate SOD isoform band was calculated by comparing the relative band intensity mm^−^^2^ to the one obtained for the Cu/Zn-SOD reference pattern of the defined activity of 4.4 U mg^−^^1^ protein (Sigma-Aldrich). Densitometric analysis was performed using Quantity1D software (Bio-Rad).

Peroxidase activity was assayed according to Nakano and Asada (1981) with some modifications of the reaction mixture, which contained 50 mM of a phosphate buffer (pH 7.0), 18 mM pyrogallol and 0.1 mM H_2_O_2_. The reaction was started by adding 0.25 ml of the enzyme extract to 0.75 ml of the reaction mixture. The oxidation of pyrogallol was measured by the decrease in absorbance at 430 nm for 1 min at 25 °C (Hitachi U-2010 spectrophotometer, Japan). The enzyme activity was quantified using the molar extinction coefficient for pyrogallol (ε = 2.47 mM^−^^1^cm^−^^1^) and the results are expressed as mM pyrogallol oxidised × min^−^^1^ × mg^−^^1^ protein.

Catalase activity was determined spectrophotometrically (Hitachi U-2010, Japan). The reaction mixture contained 50 mM of a sodium phosphate buffer (pH 7.0) and 10 mM H_2_O_2_. CAT activity was estimated by monitoring the decrease in absorbance (λ = 240 nm) that was caused by H_2_O_2_ (ε = 36 M^−^^1^ cm^−^^1^) consumption (Aebi 1984). The results are expressed as μM of consumed H_2_O_2_ × min^−^^1^ × mg^−^^1^ protein.

### Statistical analysis

Statistical analysis was performed using MS Excel 2010 (Microsoft) and Statistica 9.0 (StatSoft). All data were expressed as mean ± SD of at least four independent experiments, with two repeats for each measurement. In each sampling hour, effects of LW or JG in the presence or absence of IAA were analysed in four independent experiments, including two technical repeats of each measurement (*n* =  8), except for the interval of 0–2 h, in which only the control samples were analysed. SOD isoforms were examined in three independent experiments (isolation and PAGE analysis), including two technical repeats of each measurement (*n* =  6). Data were tested for normal distribution and variance homogeneity with the Levene's test. To compare results obtained for different variants, ANOVA and Tukey’s post hoc test were performed at *P* ≤ 0.05. Two-way ANOVA at *P* ≤ 0.05, with treatment variant and time as two predictor variables, was used to evaluate the time-dependent effects of chemicals applied.

## Results

### Hydrogen peroxide production

**Figure 1:**
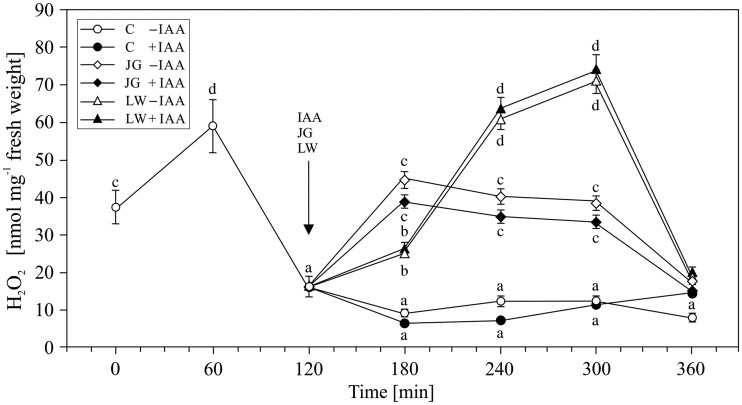
Generation of hydrogen peroxide in maize coleoptile segments exposed to juglone (JG) (50 µM) and lawsone (LW) (100 µM). Control, JG- and LW-treated coleoptiles were incubated for 4 h with or without indole-3-acetic acid at a concentration of 100 µM. The arrow indicates the moment of the application of IAA, JG and LW to the coleoptile incubation medium. The amount of hydrogen peroxide detected in the coleoptile incubation medium was expressed per fresh weight unit. Values are the means of at least four independent experiments. Bars indicate ± SDs. Means followed by the same letters are not significantly different from each other (*P* ≤ 0.05) according to the ANOVA and Tukey’s *post hoc* test.

The first step in our study was to analyse the occurrence of H_2_O_2_ in the control medium during the pre-incubation (first 2 h) and incubation (next 4 h) of the maize coleoptiles. The highest level of H_2_O_2_ (58.99 ± 2.89 nmol mg^−^^1^ fresh weight) was recorded after 1 h of the pre-incubation of the segments, after which the H_2_O_2_ concentration decreased and was maintained at a constant level (9–16 nmol mg^−^^1^ fresh weight) (Fig. 1). The 2 h pre-incubation time appeared to be long enough to prevent oxidative stress associated with preparation (cutting) of maize coleoptile segments (*F*_6,22 _= _ _136, *P* =  0.0016). The addition of IAA at a final concentration of 100 µM to the incubation medium at 2 h did not significantly affect the level of H_2_O_2_ that was determined in each of the following hours of exposure (*F*_12,44 _= _ _1.85, *P* =  0.012), though it changed with the time of incubation [**see Supporting Information—Table S1**] (*F*_12,44 _= _ _241.5, *P* =  0.038). The application of either juglone or lawsone after 2 h of the pre-incubation of a segment in the control medium resulted in an increase in H_2_O_2_ production, which was significantly higher in the presence of lawsone (*F*_10,42 _= _ _115, *P* =  0.0023) than in juglone (*F*_10,44 _= _ _67, *P* =  0.0021), regardless of the time of incubation [**see Supporting Information—Table S1**] (*F*_12,44 _= _ _3.496, *P* =  0.03 and *F*_12,44 _= _ _5.928, *P* =  0.008, respectively). The highest levels of H_2_O_2_ were detected 1 h after application for juglone (44.77 ± 2.69 nmol mg^−^^1^ fresh weight) and 2 h later for lawsone (71.00 ± 4.26 nmol mg^−^^1^ fresh weight). These maximal values declined gradually for JG and reached the control value within the next 3 h; however, they decreased rapidly for LW (Fig. 1). The addition of IAA together with juglone or lawsone did not significantly change the level of H_2_O_2_ production that was observed for JG (*F*_4,25 _= _ _2.23, *P* =  0.015) or LW (*F*_4,25 _= _ _2.13, *P* =  0.012) alone.

### Antioxidative enzyme activity

#### Superoxide dismutase

**Figure 2:**
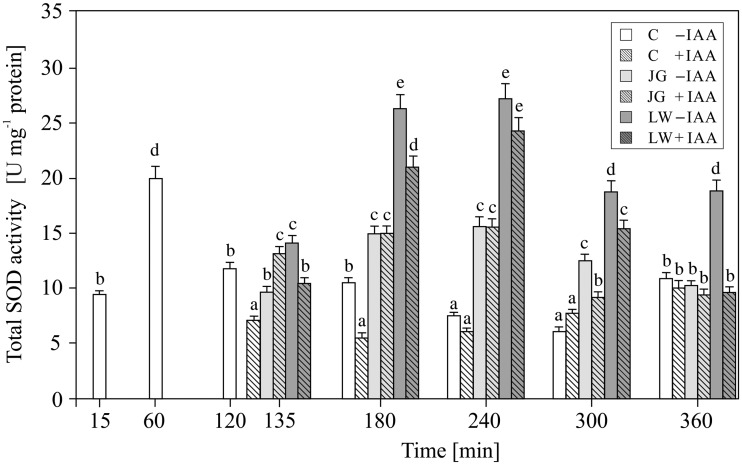
Effect of juglone (JG) and lawsone (LW) treatment on the enzymatic activity of total superoxide dismutase (SOD) in maize coleoptiles. Coleoptiles were incubated with the naphthoquinones (added after 2 h of segment pre-incubation) for 4 h, with or without auxin (100 µM). Values are the means of four independent experiments. Bars indicate ± SDs. Means followed by the same letters are not significantly different from each other (*P* ≤ 0.05) according to the ANOVA and Tukey’s *post hoc* test.

In the control maize coleoptile segments, total SOD activity ranged from 6 to 20 U mg^−^^1^ protein and depended on the duration of the experiment (Fig. 2). The highest enzymatic activity, 19.35 ± 0.97 U mg^−^^1^ protein, was detected 1 h after the preparation of the coleoptile segments; however, it decreased to 6–10 U mg^−^^1^ protein in the subsequent hours of incubation. The addition of IAA after 2 h of segment pre-incubation resulted in a decrease in SOD activity in the control samples at 3–4 h of the experiment (*F*_6,22 _= _ _56, *P* =  0.0045). Afterwards, the enzymatic activity remained at the same level (*F*_6,22 _= _ _56, *P* =  0.0045), regardless of the presence of auxin and time of incubation [**see Supporting Information—Table S1**] (*F*_6,22 _= _ _460, *P* =  0.86). After 1 and 2 h of the exposure of the coleoptile segments to JG and LW (3 and 4 h of the experiment), the activity of SOD showed a significant increase (*F*_10,42 _= _ _136, *P* =  0.0018), nearly two-fold greater in the presence of LW (*F*_4,25 _= _ _96, *P* =  0.0026) than JG (*F*_4,25 _= _ _74, *P* =  0.0035). In most cases (except for the fourth hour of the experiment), the addition of IAA together with LW caused a decrease in SOD activity (Fig. 2; *F*_4,25 _= _ _23, *P* =  0.013; **see Supporting Information—Table S1**; *F*_4,25 _= _ _1.964, *P* =  0.02), not observed if IAA was applied with JG (Fig. 2; *F*_4,25 _= _ _1.56, *P* =  0.016; **see Supporting Information—Table S1**; *F*_4,25 _= _ _3.599, *P* =  0.09).

Five SOD isoenzymes with different migration rates in the polyacrylamide gel were identified in the maize coleoptile segments [**see Supporting Information—Table S1**]. Using the KCN and H_2_O_2_ inhibition assay, two isoforms of Mn-SOD, which are referred to as Mn-SOD 1 and Mn-SOD 2, were found. We also identified three isoforms of Cu/Zn-SOD, namely Cu/Zn-SOD 1, 2 and 3. However, Fe-SOD was not found in the maize coleoptile tissue.

In the control coleoptiles, the activity of each identified isoform of Cu/Zn-SOD fell within a range of 1.05 ± 0.12 to 2.95 ± 0.18 U mg^−^^1^ protein (Table 1). As for total SOD, the highest activity of SOD isoforms was detected at 1 h of segment incubation in the control medium (*F*_18,165 _= _ _235, *P* =  0.0042 for Cu/Zn-SOD; *F*_12,112 _= _ _35, *P* =  0.0042 for Mn-SOD) and also at 4 and 6 h of incubation in the case of the Cu/Zn-SOD 3 isoform (*F*_18,165 _= _ _235, *P* =  0.0042). Both JG and LW treatment resulted in an increase in the activity of all of the Cu/Zn-SOD isoforms (*F*_42,357 _= _ _112, *P* =  0.012) (Table 1). The activity of the Cu/Zn-SOD 1 and 3 isoforms increased within 1 h of JG or LW treatment; however, the decreased in LW-treated tissues after the third hour (*F*_12,117 _= _ _35, *P* =  0.0045 and *F*_12,117 _= _ _28, *P* =  0.0036, respectively). The Cu/Zn-SOD 2 isoform exhibited an elevated although rather constant activity level in the JG-treated tissues (*F*_42,357 _= _ _112, *P* =  0.012). The highest stimulation of Cu/Zn-SOD activity was recorded for the Cu/Zn-SOD 3 isoform at 3 h exposure (*F*_6,22 _= _ _32, *P* =  0.0031). At the same time, all of the Cu/Zn-SOD isoforms displayed the greatest activity in the tissues that had been exposed to LW at 1 and 2 h (*F*_12,117 _= _ _35, *P* =  0.0045) (Table 1).

**Table 1:** Effect of juglone (JG) and lawsone (LW) treatment on the enzymatic activity of three Cu/Zn-SOD isoforms detected in 4-day-old maize coleoptile segments.

Treatment		Time (min)							
		15	60	120	135	180	240	300	360
Control	Cu/Zn-SOD 1	1.05 a	1.71 b	1.33 a		1.39 a	1.40 a	1.83 b	1.91 b
	Cu/Zn-SOD 2	1.28 a	2.95 c	1.77 b		1.86 b	1.65 b	1.98 b	1.05 a
	Cu/Zn-SOD 3	1.27 a	2.36 c	1.66 b		1.81 b	2.60 c	1.20 a	2.50 c
Juglone	Cu/Zn-SOD 1				2.44 c	4.07 d	3.28 d	2.99 c	3.47 d
	Cu/Zn-SOD 2				3.17 d	4.72 e	3.73 d	3.51 d	3.32 d
	Cu/Zn-SOD 3				2.62 c	3.38 d	3.01 c	4.29 e	2.74 c
Lawsone	Cu/Zn-SOD 1				2.59 c	4.81 e	4.60 e	1.52 a	2.64 c
	Cu/Zn-SOD 2				3.13 d	5.51 e	5.92 e	2.85 c	2.77 c
	Cu/Zn-SOD 3				3.63 d	4.65 e	4.64 e	2.84 c	3.56 d

The activity of a separate SOD isoform band was calculated by comparing the relative band intensity to the one obtained for the Cu/Zn-SOD standard of the defined activity of 4408 U mg^−1^ protein. Means followed by the same letters are not significantly different from each other (*P* ≤ 0.05) according to the ANOVA and Tukey’s *post hoc* test. Each value stands for the mean (*n* = 4).

The average activity of Mn-SODs in the control coleoptile tissues was similar to that of the Cu/Zn-SOD isoform and attained a level of 1.02 ± 0.08 to 2.87 ± 0.12 U mg^−^^1^ protein (Table 2). As in the case of the Cu/Zn-SOD isoforms, both LW and JG treatment stimulated the activity of the Mn-SOD isoforms (*F*_16,156 _= _ _67, *P* =  0.0075). In the JG-exposed coleoptiles, the Mn-SOD 1 isoform showed an increased (*F*_8,36 _= _ _22, *P* = 0.0024), though constant level, while the activity of Mn-SOD 2 fluctuated in the subsequent hours of exposure. Nevertheless, the activity of both Mn-SOD isoforms was much higher than in control tissues at 3 h of LW treatment (*F*_27,234 _= _ _144, *P* =  0.019) (Table 2).

**Table 2:** Effect of juglone (JG) and lawsone (LW) treatment on the enzymatic activity of two Mn-SOD isoforms detected in 4-day-old maize coleoptile segments.

Treatment		Time (min)							
		15	60	120	135	180	240	300	360
Control	Mn-SOD 1	2.87 d	2.81 d	1.34 a		1.02 a	1.38 a	1.72 b	1.22 a
	Mn-SOD 2	1.59 b	1.80 b	1.48 a		1.09 a	1.83 b	1.87 b	1.18 a
Juglone	Mn-SOD 1				2.24 c	2.61 c	2.02 c	2.21 c	2.26 c
	Mn-SOD 2				2.14 c	1.54 b	1.60 b	2.19 c	1.59 b
Lawsone	Mn-SOD 1				1.83 b	2.31 c	1.61 b	3.13 e	2.68 d
	Mn-SOD 2				1.90 b	2.33 c	2.68 d	3.18 e	1.99 b

The activity of a separate SOD isoform band was calculated by comparing the relative band intensity to the one obtained for the Cu/Zn-SOD standard of the defined activity of 4408 U mg^−1^ protein. Means followed by the same letters are not significantly different from each other (*P* ≤ 0.05) according to the ANOVA and Tukey’s *post hoc* test. Each value stands for the mean (*n* = 4).

### Peroxidase and catalase activity

**Figure 3:**
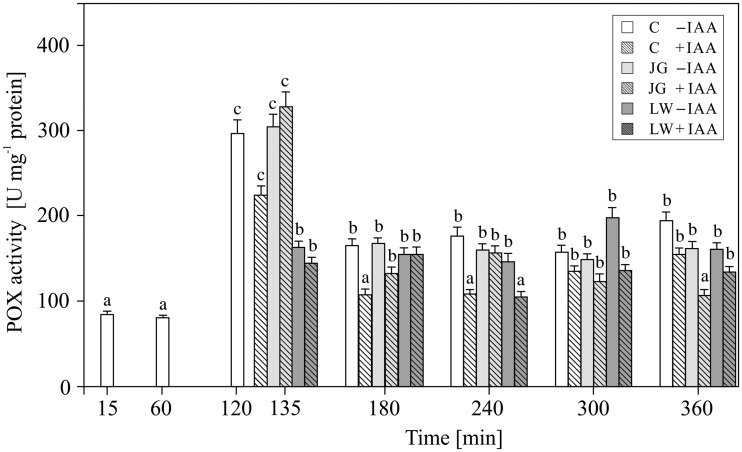
Effect of juglone (JG) and lawsone (LW) treatment on the total peroxidase (POX) activity in maize coleoptiles. Coleoptiles were incubated with the naphthoquinones (added after 2 h of segment pre-incubation) for 4 h, with or without auxin (100 µM). Values are the means of four independent experiments. Bars indicate ± SDs. Means followed by the same letters are not significantly different from each other (*P* ≤ 0.05) according to the ANOVA and Tukey’s *post hoc* test.

Peroxidases (POX) activity in the control maize coleoptile tissue increased until 2 h (*F*_6,22 _= _ _133, *P* =  0.0025) when it attained peak value of 298.91 ± 20.92 12 U mg^−^^1^ protein. As shown in Fig. 3, the application of auxin resulted in a significant decrease in the POX activity (*F*_6,22 _= _ _29, *P* =  0.0014); however, POX activity was not affected neither by JG nor LW (Fig. 3; *F*_10,42 _= _ _1.63, *P* =  0.0016), regardless of the presence of IAA in the incubation medium (**see Supporting Information—Table S1**; *F*_10,42 _= _ _85.5, *P* =  0.54 and *F*_10,42 _= _ _21.8, *P* =  0.23, respectively).

**Figure 4:**
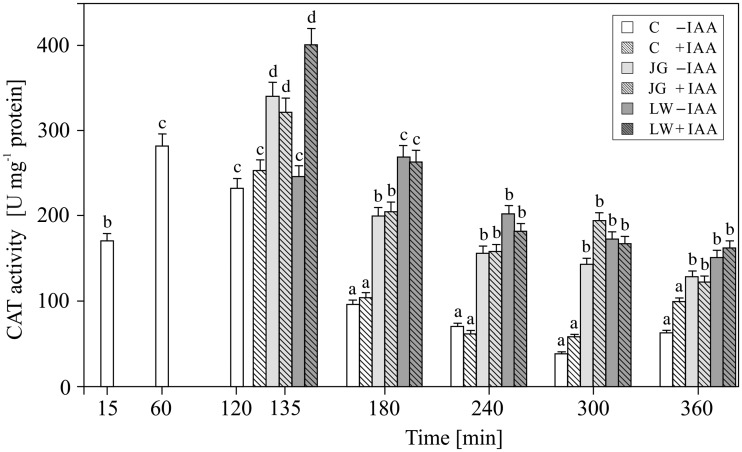
Effect of juglone (JG) and lawsone (LW) treatment on the enzymatic activity of catalase (CAT) in maize coleoptiles. Coleoptiles were incubated with the naphthoquinones (added after 2 h of segment pre-incubation) for 4 h, with or without auxin (100 µM). Values are the means of four independent experiments. Bars indicate ± SDs. Means followed by the same letters are not significantly different from each other (*P* ≤ 0.05) according to the ANOVA and Tukey’s *post hoc* test.

In the untreated maize coleoptiles, the highest CAT activity of *ca*. 300 U mg^−^^1^ protein was detected within the first 2 h, while in the subsequent hours of incubation, it decreased below 100 U mg^−^^1^ protein (*F*_6,22 _= _ _53, *P* =  0.0017) (Fig. 4). IAA applied to the incubation medium at 2 h did not significantly change CAT activity (*F*_6,22 _= _ _1.65, *P* =  0.023). The addition of both juglone and lawsone after 2 h of segment pre-incubation in the control medium resulted in an increase in CAT activity, regardless of the presence of auxin (*F*_10,42 _= _ _2.04, *P* =  0.021).

## Discussion

The obtained results indicate that both juglone and lawsone, in the presence or absence of IAA, contribute to H_2_O_2_ generation and activity of ROS scavenging enzymes (SOD, POX and CAT) in maize coleoptile segments. Previous reports evidenced both pro- and antioxidative action of quinones, depending on their chemical structure, particularly the number and arrangement of hydroxyl substituents (Liszkay *et al.* 2004; Murakami *et al.* 2010; Klotz *et al.* 2014), as well as on the dose (Babula *et al.* 2014), time of exposure (Kot *et al.* 2010) and co-presence of other factors that modulate redox homeostasis, including plant hormones, e.g. IAA (Liszkay *et al.* 2004; for a review see Tognetti *et al.* 2012). Our results indicate that the concentrations of JG and LW used here induce oxidative stress in cells of coleoptiles. This can be clearly seen in the amount of H_2_O_2_ that was generated (Fig. 1) and the elevated activity of the antioxidative enzymes (Figs 2 and 4), particularly the superoxide dismutase isoforms (Tables 1 and 2). The highest level of H_2_O_2_ recorded after 1 h of the pre-incubation of the segments in control medium probably is associated with preparation of maize coleoptile segments and is in good agreement with the growth rate kinetics of maize coleoptile segments after their excision from maize seedlings (Karcz and Burdach 2002; Karcz and Kurtyka 2007; Burdach *et al.* 2014). The total amount of H_2_O_2_ produced and the increased level of superoxide dismutase activity indicate that LW is a more efficient oxidative stress than JG. In comparison with JG, LW-treated tissues displayed a 2 h delayed and at least two-fold higher maximum H_2_O_2_ production (Fig. 1). In contrast, the studies with jack bean urease that were carried out by Kot *et al.* (2010) showed that the effectiveness of urease inhibition by juglone with reference to the quantity of generated H_2_O_2_ was 2.5 times higher than that of lawsone. Although the time required for the induction of SOD activity was similar for both quinones, the stimulation of SOD activity was much more pronounced in the LW-exposed coleoptiles. Since H_2_O_2_ is a molecule that is involved in the regulation of the activity and expression of ROS scavenging enzymes as a product of SOD activity (Alscher *et al.* 2002), and is a substrate for peroxidases and catalases (Mittler 2002), we considered the differences in the induction of SOD isoform activity as the main factor that determined the level of hydrogen peroxide. Our results indicate that the activity of catalase in maize coleoptiles is the main enzymatic mechanism that is responsible for the degradation of the H_2_O_2_ that is generated under the oxidative stress induced by JG or LW (Fig. 4). We found that exposure to both quinones resulted in at least *ca.* a two-fold stimulation of catalase activity, regardless of the time of exposure or the type of quinone applied. We also observed that, except for the moment of the application of JG and LW, peroxidase activity was not involved in the neutralisation of H_2_O_2_ (Fig. 3). It can either be assumed that the enzyme did not participate in H_2_O_2_ scavenging or that it was inhibited by an elevated H_2_O_2_ concentration (Hema *et al.* 2007). We would rather explain the lack of an effect of LW or JG on the peroxidase activity with its H_2_O_2_-mediated inhibition rather than with direct LW or JG action. Some experimental data indicate a greater resistance of catalase to high H_2_O_2_ concentrations (Asada 1999; Alscher *et al.* 2002). In the present study, we identified five SOD isoforms (Cu/Zn-SOD 1, 2 and 3 as well as Mn-SOD 1 and 2) in maize coleoptile segments. However, we did not determine any Fe-SOD isoforms, typically assigned to chloroplasts (Scandalios 1993; Allen *et al.* 2007) [**see Supporting Information—Figure S1**]. Such an observation probably results from the fact that coleoptile segments were excised from etiolated maize seedlings. All five of the SOD isoforms that were identified in the examined tissue responded to JG- and LW-induced stress. Generally, the Cu/Zn-SOD isoforms attained the highest enzymatic activity within the first 2 h of exposure to quinones; afterwards, the activity gradually decreased to a level only slightly higher than in the control cells. Cu/Zn-SOD is primarily considered to be a cytosolic enzyme (Allen *et al.* 2007); however, because it is also present in the chloroplasts and cell wall space, we believe it is reasonable to assume that both JG and LW induce oxidative stress in the cytosolic or cell wall spaces of maize coleoptiles. In contrast to Cu/Zn-SOD, the highest recorded activity of mitochondrial Mn-SOD isoforms was observed at 2 and 3 h of coleoptile exposure to JG and LW with effects of LW appearing to be more pronounced in comparison with JG. Since mitochondrial activity is assumed to be the only source of metabolic energy in the tissue of an etiolated coleoptile (Hager 2003), the elevated activity of Mn-SOD isoforms, which are associated with the mitochondrial space, can be expected. Among all of the SODs, Mn-SOD displayed the highest resistance to the increased concentrations of H_2_O_2_ (Allen *et al.* 2007). Moreover, due to the fact that the cytosolic space may be the site of the modification of JG and LW (Sharma *et al.* 2009) or their cross-reaction with glutathione (Sytykiewicz 2011), the mitochondrial space might be less susceptible to JG- or LW-mediated oxidative stress (Hejl *et al.* 1993). Therefore, it is not surprising that the induction of this isoform activity is delayed in comparison to the cytosol. On the other hand, since both quinones may potentially interact with the compounds of the mitochondrial electron transport chain (particularly substitute other quinones), a mitochondrion is likely to be expected as the main target action site of naphthoquinones.

According to Schopfer *et al.* (2002), the coleoptile growth of maize seedlings is accompanied by the release of reactive oxygen intermediates in the cell wall. These authors found that auxin promoted the release of O_2_^−^ and the subsequent generation of ˙OH when elongation growth is induced by IAA. Moreover, their experimental data indicated that the generation of ˙OH in the cell wall causes an increase in the wall extensibility *in vitro* and replaces auxin in the induction of growth. It was also proposed that auxin not only induces cell elongation but is also involved in the tolerance to oxidative stress (for a review see Tognetti *et al.* 2012). On the one hand, auxin can modulate ROS homeostasis by regulating H_2_O_2_ levels (Iglesias *et al.* 2010), inducing ROS detoxification enzymes (Laskowski *et al.* 2002) and interacting with other hormonal signalling networks (Sun *et al.* 2009; De Tullio *et al.* 2010). On the other hand, the response and sensitivity to auxin are affected by the redox state (Bashandy *et al.* 2010). For example, an increase in ROS levels can affect auxin biosynthesis (Woodward and Bartel 2005), transport (Grunewald and Friml 2010) and distribution (Pasternak *et al.* 2005; Santelia *et al.* 2008). Here, in most cases, presence of auxin did neither affect the activity of antioxidative enzymes nor production of hydrogen peroxide. In the presented experimental model, we also did not establish any relationships between the effects of JG or LW and presence of the applied auxin. Such an observation may be associated with the ability of JG (probably of LW as well) to inhibit the auxin-dependent interaction between Aux/IAA proteins (family of transcriptional regulators) and the SCF-TIR1 complex, which in turn blocks auxin activity (Dharmasiri *et al.* 2003).

## Conclusions

To conclude, we found that LW was a more effective oxidative stress inducer in maize coleoptile segments than JG. It was found that JG- and LW-mediated changes in the activity of Cu/Zn-SOD isoenzymes are the main factors that determine the amount of H_2_O_2_ that is generated in tissues that are exposed to both quinones. The cell potential to neutralise hydrogen peroxide, which is determined by the POX and CAT activity, points to the activity of catalase as the main enzymatic mechanism responsible for the degradation of the H_2_O_2_ that is generated under the oxidative stress that is induced by JG or LW in maize coleoptiles. The results presented here also indicate that IAA does not play any role in JG- and LW-induced oxidative stress in maize coleoptiles.

## Supplementary Material

Supplementary Data

## Supporting Information

The following additional information is available in the online version of this article —

**Figure S1.** SOD isoenzyme profile obtained for control 4-day-old etiolated maize (*Zea mays* L.) coleoptiles incubated with or without IAA (0.1 mM) for 4 h.

**Table S1.** A set of results of two-way ANOVA analysiswith treatment variant and time of exposition as the two predictor variables, performed to evaluate the time dependent effects of chemicals applied on the hydrogen peroxide production and antioxidative enzymes activity in themaize coleoptiles.
