# Supplementary material for: Effects of juglone and lawsone on oxidative stress in maize coleoptile cells treated with IAA
Source: AoB Plants. 2016 Nov 17;8:plw073. doi: 10.1093/aobpla/plw073 (PMC5199135; doi:10.1093/aobpla/plw073)
Supplement: Supplementary Data [file supp_8_plw073_index.html]

Effects of juglone and lawsone on oxidative stress in maize coleoptile cells treated with IAA — Effects of juglone and lawsone on oxidative stress in maize coleoptile cells treated with IAA — Supplementary Data 

# Effects of juglone and lawsone on oxidative stress in maize coleoptile cells treated with IAA

## Supplementary Data

files

- Supplementary Data - docx file
