# Supplementary material for: Effects of juglone and lawsone on oxidative stress in maize coleoptile cells treated with IAA
Source: AoB Plants. 2016 Nov 17;8:plw073. doi: 10.1093/aobpla/plw073 (PMC5199135; doi:10.1093/aobpla/plw073)
Supplement: Supplementary Data [file supp_plw073_Supporting_information.docx]

**Supporting information**

**Fig. S1**


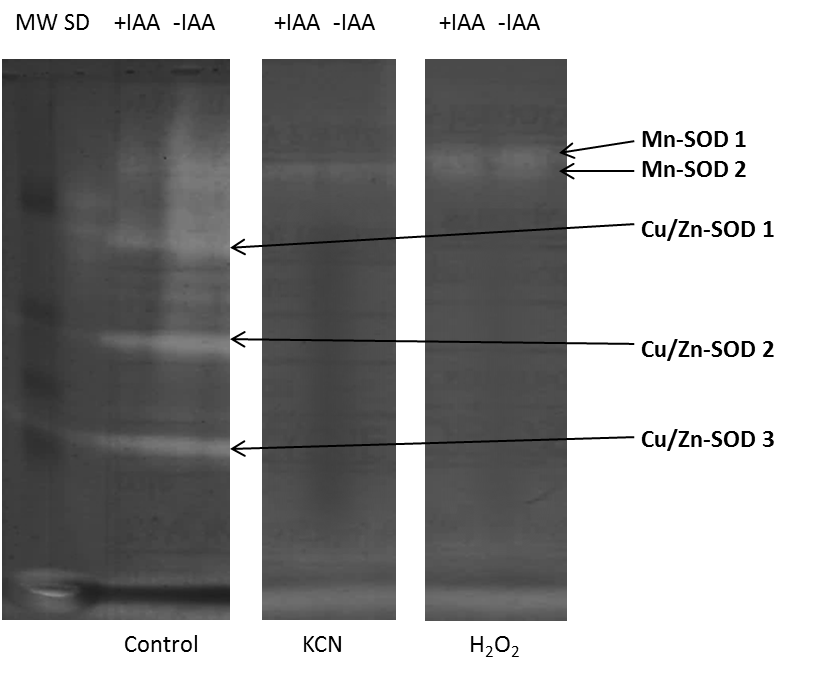


**SOD isoenzyme profile obtained for control 4-day-old etiolated maize (*Zea mays* L.) coleoptiles incubated with or without IAA (0.1 mM) for 4 h.** PAGE analysis in a 12.5% gel stained for SOD activity according to the method of Beauchamp and Fridovich (1971), involving reduction of NBT. SOD isoforms were identified according to their different sensitivity to H_2_O_2_ and KCN. Cu/Zn-SOD was inhibited by CN^-^ and inactivated by H_2_O_2_, Fe-SOD was resistant to CN^-^ and sensitive to H_2_O_2_, while Mn-SOD was resistant to both CN^-^ and H_2_O_2_. For inhibition assays, gels were incubated in 2 mM KCN or 5 mM H_2_O_2_ prior to staining. 50μg protein per line; MwM – molecular weight marker, SOD M – Cu/Zn-SOD reference pattern of defined activity of 4408 U/mg (Sigma-Aldrich).

**Table S1**

A set of results of Two-way ANOVA analysis with treatment variant and time of exposition as the two predictor variables, performed to evaluate the time dependent effects of chemicals applied on the hydrogen peroxide production and antioxidative enzymes activity in the maize coleoptiles.

Hydrogen peroxide production

|  | SS | df | MS | F | p |
| --- | --- | --- | --- | --- | --- |
| IAA | 23.41159 | 1 | 23.41159 | 48.47707 | 0.42101 |
| Time | 466.4943 | 4 | 116.6236 | 241.4859 | 0.03869 |
| IAA*Time | 310.9359 | 4 | 77.73397 | 160.9594 | 0.06794 |
| err. | 172.9877 | 70 | 2.141106 |  |  |
|  |  |  |  |  |  |
|  | SS | df | MS | F |  |
| JG | 8140.361 | 1 | 8140.361 | 3496.547 | 0.03057 |
| Time | 2627.439 | 4 | 656.8599 | 282.1425 | 0.01148 |
| JG*Time | 3053.016 | 4 | 763.2539 | 327.8422 | 0.02830 |
| err. | 153.389 | 70 | 2.627 |  |  |
|  |  |  |  |  |  |
|  | SS | df | MS | F |  |
| LW | 13802.88 | 1 | 13802.88 | 5928.783 | 0.00871 |
| Time | 11342.18 | 4 | 2835.545 | 1217.958 | 0.00851 |
| LW*Time | 10840.03 | 4 | 2710.007 | 1164.035 | 0.04040 |
| err. | 162.968 | 70 | 2.328114 |  |  |
|  |  |  |  |  |  |
|  |  |  |  |  |  |
|  | SS | df | MS | F |  |
| JG+IAA | 5751.56 | 1 | 5751.56 | 3243.944 | 0.03314 |
| Time | 998.2718 | 4 | 249.568 | 140.7591 | 0.01252 |
| JgG+IAA* Time | 3527.068 | 4 | 881.767 | 497.3264 | 0.01512 |
| err. | 124.111 | 70 | 1.773015 |  |  |
|  |  |  |  |  |  |
|  | SS | df | MS | F | p |
| LW+IAA | 14963.22 | 1 | 14963.22 | 15.05452 | 0.04700 |
| Time | 9954.493 | 4 | 2488.623 | 2.503809 | 0.04992 |
| LW+IAA* Time | 12397.87 | 4 | 3099.468 | 3.118381 | 0.02024 |
| err. | 69575.45 | 70 | 993.935 |  |  |

Total Superoxide dismutase activity

|  | SS | df | MS | F | p |
| --- | --- | --- | --- | --- | --- |
| IAA | 84.75207 | 1 | 84.75207 | 460.2614 | 0.86799 |
| Time | 167.4911 | 4 | 41.87278 | 227.3977 | 0.028542 |
| IAA*Time | 125.651 | 4 | 31.41274 | 170.5926 | 0.906961 |
| err. | 12.88973 | 70 | 0.184139 |  |  |
|  |  |  |  |  |  |
|  | SS | df | MS | F | p |
| JG | 210.4541 | 1 | 210.4541 | 658.0943 | 0.038586 |
| Time | 102.0051 | 4 | 25.50129 | 79.74302 | 0.00385 |
| JG*Time | 322.1863 | 4 | 80.54657 | 251.8707 | 0.035034 |
| err. | 22.38553 | 70 | 0.319793 |  |  |
|  |  |  |  |  |  |
|  | SS | df | MS | F | p |
| LW | 2752.083 | 1 | 2752.083 | 3930.138 | 0.00825 |
| Time | 438.5243 | 4 | 109.6311 | 156.5596 | 0.009946 |
| LW*Time | 741.4343 | 4 | 185.3586 | 264.703 | 0.037988 |
| err. | 49.01758 | 70 | 0.700251 |  |  |
|  |  |  |  |  |  |
|  | SS | df | MS | F | p |
| JG+IAA | 533.736 | 1 | 533.736 | 1964.472 | 0.025593 |
| Time | 47.41827 | 4 | 11.85457 | 43.63199 | 0.013734 |
| JG+IAA* Time | 341.4681 | 4 | 85.36702 | 314.2023 | 0.047242 |
| err. | 19.01861 | 70 | 0.271694 |  |  |
|  |  |  |  |  |  |
|  | SS | df | MS | F | p |
| LW+IAA | 1570.75 | 1 | 1570.75 | 3599.898 | 0.09259 |
| Time | 425.3037 | 4 | 106.3259 | 243.6813 | 0.032997 |
| LW+IAA* Time | 1007.274 | 4 | 251.8184 | 577.1258 | 0.097057 |
| err. | 30.54323 | 70 | 0.436332 |  |  |

Superoxide dismutase isoforms activities

| Mn-SOD 1 | |  |  |  |  |
| --- | --- | --- | --- | --- | --- |
|  |  |  |  |  |  |
|  | SS | df | MS | F | p |
| JG | 13.02833 | 1 | 13.02833 | 1482.676 | 0.014673773 |
| Time | 0.482638 | 4 | 0.120659 | 13.73152 | 0.02347397 |
| JG*Time | 2.193716 | 4 | 0.548429 | 62.41338 | 0.033140632 |
| err. | 0.439352 | 50 | 0.008787 |  |  |
|  |  |  |  |  |  |
|  | SS | df | MS | F | p |
| LW | 14.24327 | 1 | 14.24327 | 1520.678 | 0.026816661 |
| Time | 6.775621 | 4 | 1.693905 | 180.8492 | 0.035350135 |
| LW*Time | 3.965529 | 4 | 0.991382 | 105.8446 | 0.437623749 |
| err. | 0.46832 | 50 | 0.009366 |  |  |
|  |  |  |  |  |  |
| Mn-SOD2 |  |  |  |  |  |
|  |  |  |  |  |  |
|  | SS | df | MS | F | p |
| JG | 10.14526 | 1 | 10.14526 | 3.339966 | 0.035865099 |
| Time | 5.927518 | 4 | 1.481879 | 0.487856 | 0.044569631 |
| JG*Time | 6.32713 | 4 | 1.581782 | 0.520746 | 0.020856545 |
| err. | 151.8767 | 50 | 3.037534 |  |  |
|  |  |  |  |  |  |
|  | SS | df | MS | F | p |
| LW | 12.92369 | 1 | 12.92369 | 1232.112 | 0.010018383 |
| Time | 8.185529 | 4 | 2.046382 | 195.0969 | 0.039787146 |
| LW*Time | 1.589025 | 4 | 0.397256 | 37.8734 | 0.018531451 |
| err. | 0.524453 | 50 | 0.010489 |  |  |

| Cu/Zn-SOD1 | |  |  |  |  |
| --- | --- | --- | --- | --- | --- |
|  |  |  |  |  |  |
|  | SS | df | MS | F | p |
| JG | 42.26118 | 1 | 42.26118 | 2525.017 | 0.005826506 |
| Time | 5.561092 | 4 | 1.390273 | 83.0659 | 0.049200203 |
| JG*Time | 4.957319 | 4 | 1.23933 | 74.04736 | 0.032555704 |
| err. | 0.836849 | 50 | 0.016737 |  |  |
|  |  |  |  |  |  |
|  | SS | df | MS | F | p |
| LW | 41.28816 | 1 | 41.28816 | 2263.628 | 0.003889225 |
| Time | 19.08078 | 4 | 4.770196 | 261.5266 | 0.04383986 |
| LW*Time | 31.28485 | 4 | 7.821212 | 428.7989 | 0.016745282 |
| err. | 0.911991 | 50 | 0.01824 |  |  |
|  |  |  |  |  |  |
|  |  |  |  |  |  |
| Cu/Zn-SOD 2 | |  |  |  |  |
|  |  |  |  |  |  |
|  | SS | df | MS | F | p |
| JG | 61.82591 | 1 | 61.82591 | 2947.203 | 0.003317781 |
| Time | 7.946848 | 4 | 1.986712 | 94.70534 | 0.034013324 |
| JG*Time | 4.160914 | 4 | 1.040228 | 49.58705 | 0.039866977 |
| err. | 1.048891 | 50 | 0.020978 |  |  |
|  |  |  |  |  |  |
|  | SS | df | MS | F | p |
| LW | 103.9478 | 1 | 103.9478 | 3646.049 | 0.00986472 |
| Time | 31.00522 | 4 | 7.751304 | 271.8828 | 0.04516745 |
| LW*Time | 17.62087 | 4 | 4.405217 | 154.5163 | 0.025157011 |
| err. | 1.425486 | 50 | 0.02851 |  |  |
|  |  |  |  |  |  |
|  |  |  |  |  |  |
| Cu/Zn-SOD 3 | |  |  |  |  |
|  |  |  |  |  |  |
|  | SS | df | MS | F | p |
| JG | 23.60047 | 1 | 23.60047 | 1279.625 | 0.023954044 |
| Time | 3.25271 | 4 | 0.813177 | 44.09073 | 0.047911576 |
| JG*Time | 15.89816 | 4 | 3.97454 | 215.5008 | 0.045175627 |
| err. | 0.922164 | 50 | 0.018443 |  |  |
|  |  |  |  |  |  |
|  | SS | df | MS | F | p |
| LW | 54.7857 | 1 | 54.7857 | 2244.645 | 0.002101405 |
| Time | 17.87555 | 4 | 4.468888 | 183.0965 | 0.036092923 |
| LW*Time | 5.022025 | 4 | 1.255506 | 51.43982 | 0.004503677 |
| err. | 1.220364 | 50 | 0.024407 |  |  |

Peroxidase activity

|  | SS | df | MS | F | p |
| --- | --- | --- | --- | --- | --- |
| IAA | 56913.44 | 1 | 56913.44 | 354.9223 | 0.003526 |
| Time | 174042.2 | 4 | 43510.56 | 271.3395 | 0.009394 |
| IAA*Time | 7263.717 | 4 | 1815.929 | 11.32446 | 0.039288 |
| err. | 11224.83 | 70 | 160.3547 |  |  |
|  |  |  |  |  |  |
|  | SS | df | MS | F | p |
| JG | 56913.44 | 1 | 56913.44 | 285.4086 | 0.803044 |
| Time | 174042.2 | 4 | 43510.56 | 218.1961 | 0.025752 |
| JG*Time | 7263.717 | 4 | 1815.929 | 9.106493 | 0.034227 |
| err. | 13958.73 | 70 | 199.4104 |  |  |
|  |  |  |  |  |  |
|  | SS | df | MS | F | p |
| LW | 22977.47 | 1 | 22977.47 | 134.082 | 0.02657 |
| Time | 52224.6 | 4 | 13056.15 | 76.18746 | 0.013209 |
| LW*Time | 66904.25 | 4 | 16726.06 | 97.60276 | 0.047962 |
| err. | 11995.81 | 70 | 171.3687 |  |  |
|  |  |  |  |  |  |
|  | SS | df | MS | F | p |
| JG+IAA | 12327.77 | 1 | 12327.77 | 85.5158 | 0.549885 |
| Time | 285011.4 | 4 | 71252.86 | 494.2696 | 0.04658 |
| JG+IAA* Time | 53664.72 | 4 | 13416.18 | 93.0659 | 0.084172 |
| err. | 10091.05 | 70 | 144.1579 |  |  |
|  |  |  |  |  |  |
|  | SS | df | MS | F | p |
| LW+IAA | 2233.584 | 1 | 2233.584 | 21.85188 | 0.02379 |
| Time | 52426.47 | 4 | 13106.62 | 128.2263 | 0.038799 |
| LW+IAA* Time | 34507.39 | 4 | 8626.846 | 84.39925 | 0.028283 |
| err. | 7155.031 | 70 | 102.2147 |  |  |

Catalase activity

|  | SS | df | MS | F | p |
| --- | --- | --- | --- | --- | --- |
| IAA | 4778.477 | 1 | 4778.477 | 80.22554 | 0.534034 |
| Time | 389608 | 4 | 97402 | 1635.276 | 0.009894 |
| IAA*Time | 4446.128 | 4 | 1111.532 | 18.66144 | 0.051606 |
| err. | 4169.413 | 70 | 59.56304 |  |  |
|  |  |  |  |  |  |
|  | SS | df | MS | F | p |
| JG | 175780.9 | 1 | 175780.9 | 1681.285 | 0.960029 |
| Time | 421805.5 | 4 | 105451.4 | 1008.607 | 0.029452 |
| JG*Time | 4972.218 | 4 | 1243.055 | 11.88939 | 0.040455 |
| err. | 7318.609 | 70 | 104.5516 |  |  |
|  |  |  |  |  |  |
|  | SS | df | MS | F | p |
| LW | 236209.1 | 1 | 236209.1 | 2183.433 | 0.968936 |
| Time | 208436.8 | 4 | 52109.2 | 481.6789 | 0.006494 |
| LW*Time | 59015.56 | 4 | 14753.89 | 136.3797 | 0.886275 |
| err. | 7572.77 | 70 | 108.1824 |  |  |
|  |  |  |  |  |  |
|  | SS | df | MS | F | p |
| JG+IAA | 145648.4 | 1 | 145648.4 | 1281.732 | 0.948215 |
| Time | 356607.2 | 4 | 89151.8 | 784.5519 | 0.019782 |
| JG+IAA* Time | 27998.25 | 4 | 6999.562 | 61.59741 | 0.077875 |
| err. | 7954.383 | 70 | 113.634 |  |  |
|  |  |  |  |  |  |
|  | SS | df | MS | F | p |
| LW+IAA | 287399.9 | 1 | 287399.9 | 1951.163 | 0.965366 |
| Time | 508479.9 | 4 | 127120 | 863.0196 | 0.012537 |
| LW+IAA* Time | 22255.92 | 4 | 5563.979 | 37.77395 | 0.06834 |
| err. | 10310.77 | 70 | 147.2967 |  |  |
